# Dengue Fever, Crimean-Congo Hemorrhagic Fever, and COVID-19 Triple Co-infection: Out of the Frying Pan Into the Fire

**DOI:** 10.7759/cureus.29028

**Published:** 2022-09-11

**Authors:** Fawad Rahim, Said Amin, Mohammad Noor, Barkat Ali, Azhar Wahab

**Affiliations:** 1 Internal Medicine, Khyber Girls Medical College, Peshawar, PAK; 2 Internal Medicine, Hayatabad Medical Complex Peshawar, Peshawar, PAK; 3 Internal Medicine, Khyber Girls Medical College , Peshawar, PAK

**Keywords:** dengue fever, public health, covid-19, coinfection, sars-cov-2, crimean-congo hemorrhagic fever virus, dengue virus

## Abstract

In developing countries, infectious diseases are thriving due to poor hygiene, inadequate public health infrastructure, and socio-cultural factors. Generally, infections are due to a single pathogen, but due to the shared risk factors for transmission, co-infections are not uncommon. The severity and outcome of infections are adversely affected by co-infection. Co-infections present as diagnostic and therapeutic enigmas because of the complex interaction between different pathogens involved and distorted host responses. The southeast Asian region, particularly Pakistan, is known for unique combinations of different infections.

We present a distinctive case of triple co-infection of dengue virus, Crimean-Congo hemorrhagic fever virus, and severe acute respiratory syndrome coronavirus-2. The index case was a 60-year-old gentleman who presented with fever, cough, shortness of breath, bruises, and hemoptysis. He had thrombocytopenia, deranged liver and renal function, coagulopathy, and infiltrates in both lung fields. Subsequent investigations revealed a positive polymerase chain reaction for ribonucleic acid of dengue virus, Crimean-Congo Hemorrhagic fever virus, and severe acute respiratory syndrome coronavirus-2. He received supportive treatment including antibiotics, blood products, ribavirin, and supplemental oxygen. He developed multi-organ failure and succumbed to the triple co-infection. This case will act as a wake-up call for clinicians, public health authorities, and infectious disease specialists to plan before the volcano of co-infections erupts.

## Introduction

In developing countries like Pakistan, poverty, illiteracy, poor hygienic conditions, and poor infrastructure of public health are responsible for the increased prevalence of infectious diseases. The infections not only occur as a single illness but also as co-infections. Globally, 16% of infections occur as co-infections [1]. The presence of an ongoing pandemic of SARS-CoV-2 and the local outbreaks of other infectious diseases increase the chances of co-infections [2]. Hickam's dictum approach and better diagnostic facilities amplify the higher reporting of co-infections [3]. Hence the prevalence of co-infections has increased to 19% in the era of COVID-19 [4]. The risk of co-infections is increased by seasonal timing, sociocultural factors, the natural history of each infection, and shared risk factors [5].

Even if co-infection is just a stroke of bad luck, the adverse effects on health might be expected to be additive. The complex interaction between the pathogens, altered host responses, changes in the virulence of pathogens, and diagnostic and therapeutic complexities are the reasons for the worse outcomes in the presence of co-infections [6].

Pakistan, a developing country located in the sub-tropics, experiences seasonal outbreaks [7]. On top of that, the above-stated predisposing factors place Pakistan on the list of the World Health Organization (WHO) high infectious disease burden regions [8]. With frequent outbreaks of infectious diseases, this region is known for interesting and rare combinations of co-infections [9]. We report an extraordinary case of a 60-year-old man with triple viral co-infections, including one tick-borne (Crimean-Congo hemorrhagic fever (CCHF) virus), one mosquito-borne (Dengue virus), and one droplet-transmitted (SARS-CoV-2) infection, which has not been reported before in the literature.

## Case presentation

A 60-year-old gentleman presented to the emergency room on July 28, 2022, with complaints of cough and shortness of breath for seven days, fever for five days, and confusion for one day. The cough was productive, bringing up greenish sputum tinged with frank blood. The shortness of breath was gradual in onset but progressed to New York Heart Association class 4 over time. The fever was high grade, intermittent, and responded only to paracetamol. It was associated with generalized body aches and fatigue. He gradually became confused a day before he was referred to Hayatabad Medical Complex, Peshawar, Pakistan. He received ceftriaxone 2 grams daily and paracetamol for five days at the local healthcare facility before the referral. Before referral, he remained admitted for two days in a local health care facility where he was diagnosed and managed as a case of dengue fever based on a positive dengue NS-1 antigen test. He received four units of platelets in addition to supportive treatment during the hospital stay.

He reports having bronchial asthma since a young age. He used inhaled bronchodilators and corticosteroids off and on, resulting in poor control of his asthma. The rest of his medical and surgical histories are unremarkable. He was a manual laborer by profession. He did not report allergies to any medications and did not have recent ill contacts. He contributed to the religious slaughtering of animals during the recently celebrated Eid ul Adha festival.

**Figure 1:**
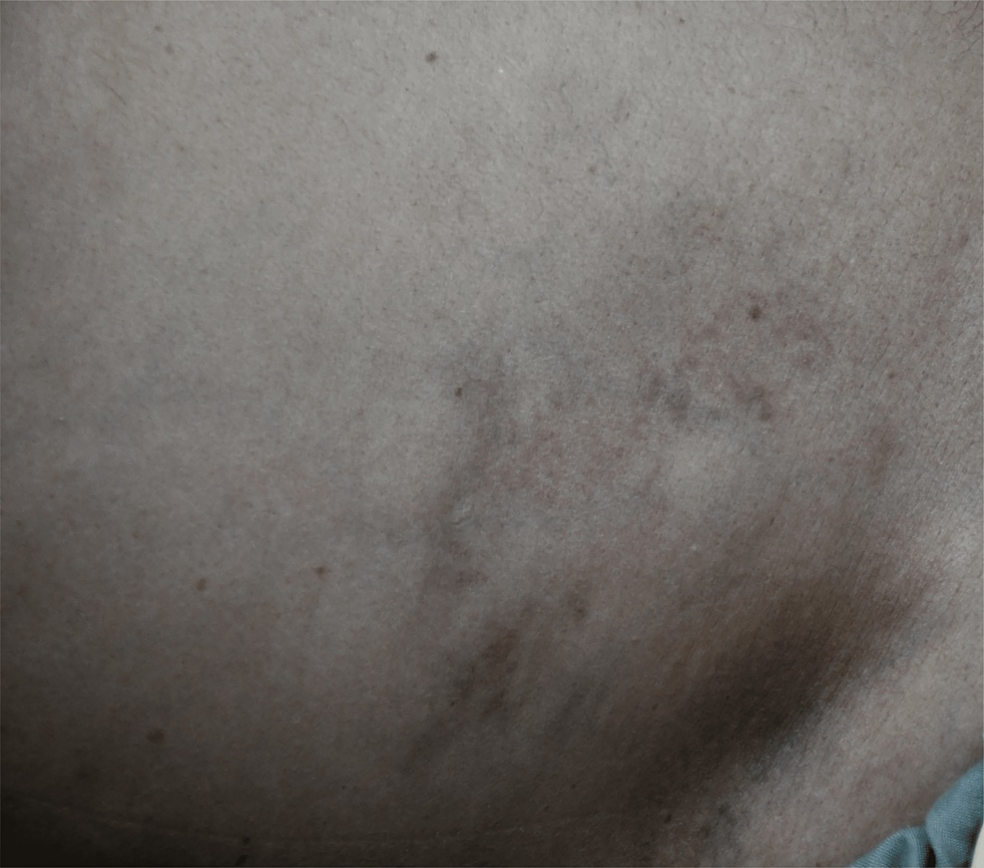
Picture of the right flank of the patient showing bruising.

**Figure 2:**
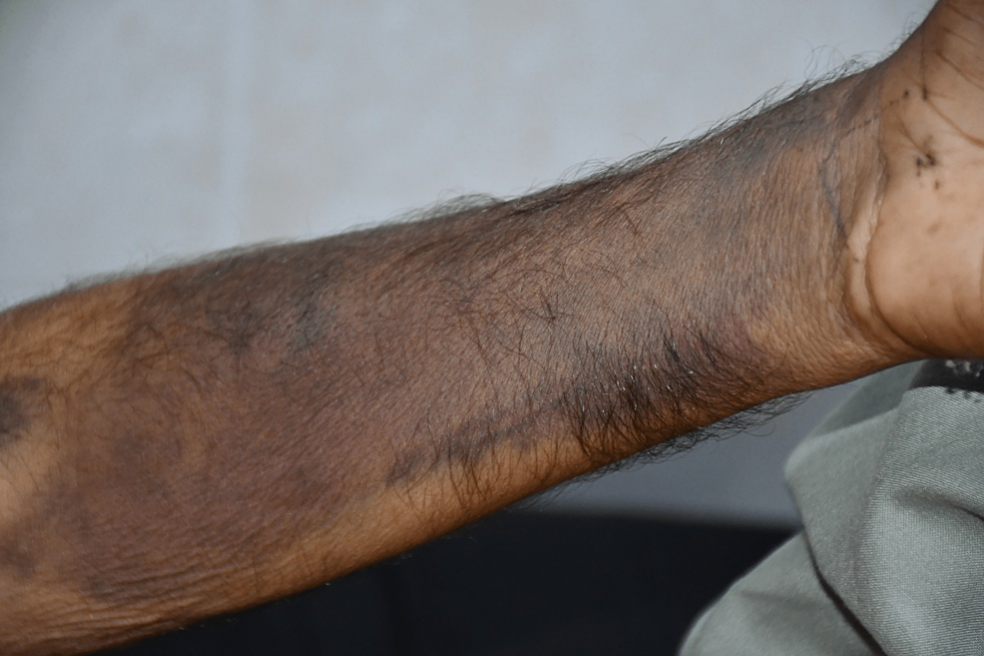
Picture of the left forearm of the patient showing ecchymosis.

Examination revealed a temperature of 101°F, a pulse of 104 beats per minute, blood pressure of 100/60 mmHg, respiratory rate of 26 breaths per minute, and oxygen saturation of 92% on room air. He had bruises on both hands and forearms, a bruise on the right flank, and pitting pedal edema limited to the feet (Figure 1, Figure 2).

He had bilateral coarse crackles and wheezes on chest examination. The neck veins were not distended. His Glasgow coma scale was 13/15, the neck was supple, pupils were equal and reactive, there was no focal weakness and plantar responses were flexor. He was admitted to the isolation unit for further management.

A differential diagnosis of community-acquired/atypical pneumonia, COVID-19, pulmonary/disseminated tuberculosis, acute severe asthma, dengue fever with complications, and CCHF was considered. He was started on cefoperazone-sulbactam, clarithromycin, hydrocortisone, supplemental oxygen with a target saturation of 94%, paracetamol, and nebulization with salbutamol and ipratropium bromide. Results of the investigations performed on the day of admission are summarized in Table 1.

**Table 1:** Results of investigations at the time of admission. g/dL: Gram/deciliter, mcL: Microliter, mg/dL: milligram/deciliter, IU/L: International unit/liter, PT: Prothrombin time, APTT: Activated partial thromboplastin time, ng/ml: Nanogram/milliliter, NS-1: Non-structural-1, IgM: immunoglobulin M,  g/dL: Gram/deciliter, U/L: Unit/liter, mmol/L: mmHg: Millimeter of mercury, mmol/L: Millimole/liter, ECG: Electrocardiogram

Investigations	Reference range	Results
Hemoglobin (g/dL)	13.5 – 17.5	9.1
Platelet count (x10^3^/mcL)	150 – 450	22
White cell count (x10^3^/mcL)	4.5 – 11	8,600
Neutrophils (%)	40 – 60 %	78
Lymphocytes (%)	20 – 40 %	13
Monocytes (%)	2 – 8 %	8
Eosinophils (%)	1 – 4 %	1
Total bilirubin (mg/dL)	0.2 – 1.2	0.7
Alanine aminotransferase (IU/L)	< 45	244
Alkaline phosphatase (IU/L)	30 – 120	110
Urea (mg/dL)	18 – 45	82
Creatinine (mg/dL)	0.6 – 1.2	1.3
C-reactive protein (mg/dL)	< 0.5	34.8
Ferritin (ng/ml)	30 – 400	2,000
Serum albumin (g/dL)	3.5 – 5.5	1.9
Serum glucose (mg/dL)	70 – 140	142
Serum calcium (mg/dL)	8 – 10	7.9
Serum lipase (U/L)	0 – 160	88
Serum amylase (U/L)	40 – 120	96
PT (seconds)	12	36
APTT (seconds)	28	> 60
Troponin I (ng/ml)	< 0.6	0.11
Dengue NS-1 antigen	Negative	Positive
Dengue IgM antibodies	Non-Reactive	Reactive
Urinalysis	Normal	
Malarial parasite	Not seen	
Arterial blood gases	Ph = 7.4, PaCO­_2 _= 31 mmHg, PaO_2 _= 120 mmHg, HCO_3 _= 21 mmol/L	
ECG	Sinus tachycardia	
Echocardiogram	Ejection fraction = 58%, diastolic dysfunction, mild tricuspid regurgitation	

**Figure 3:**
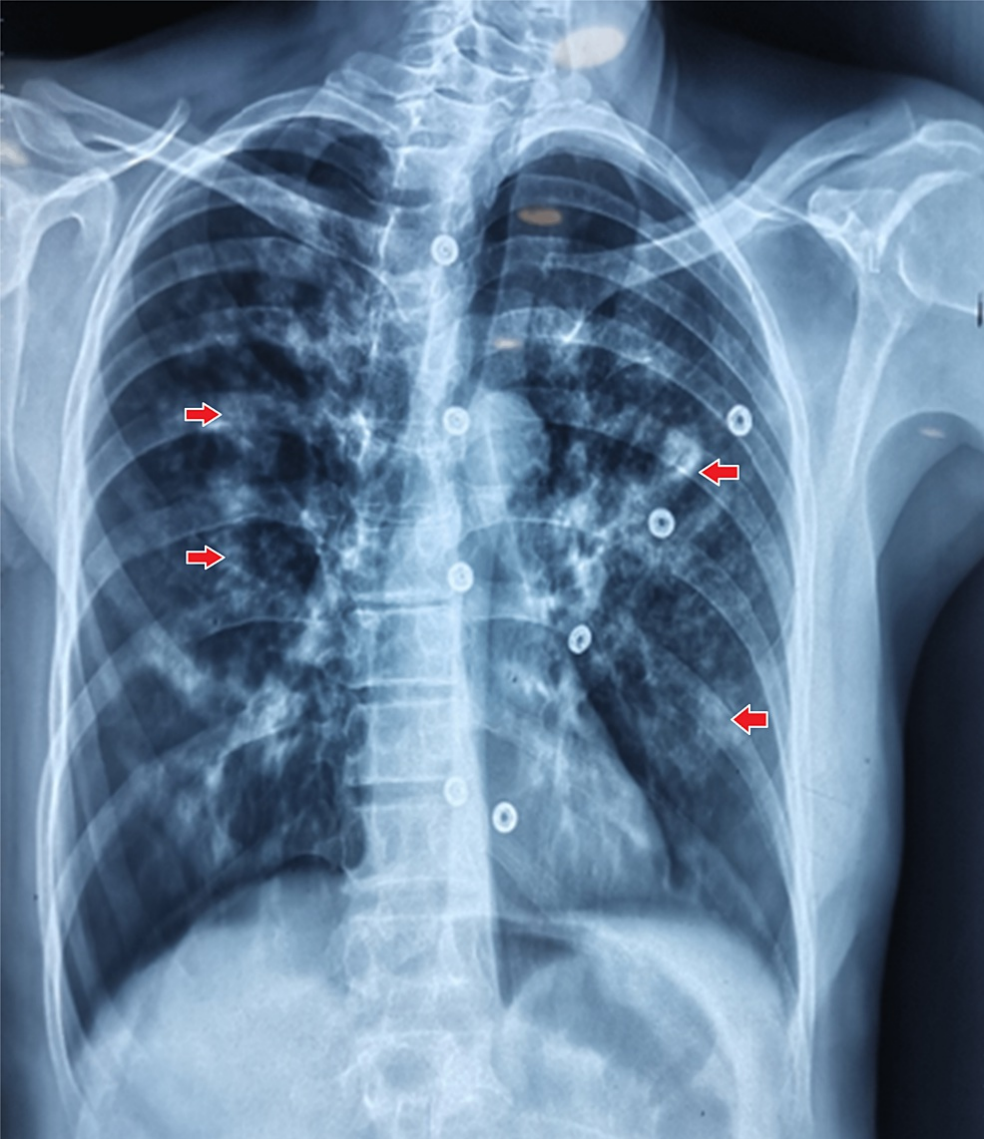
X-ray chest (Posterior anterior view) showing multifocal infiltrates (arrows) involving both lung fields.

**Figure 4:**
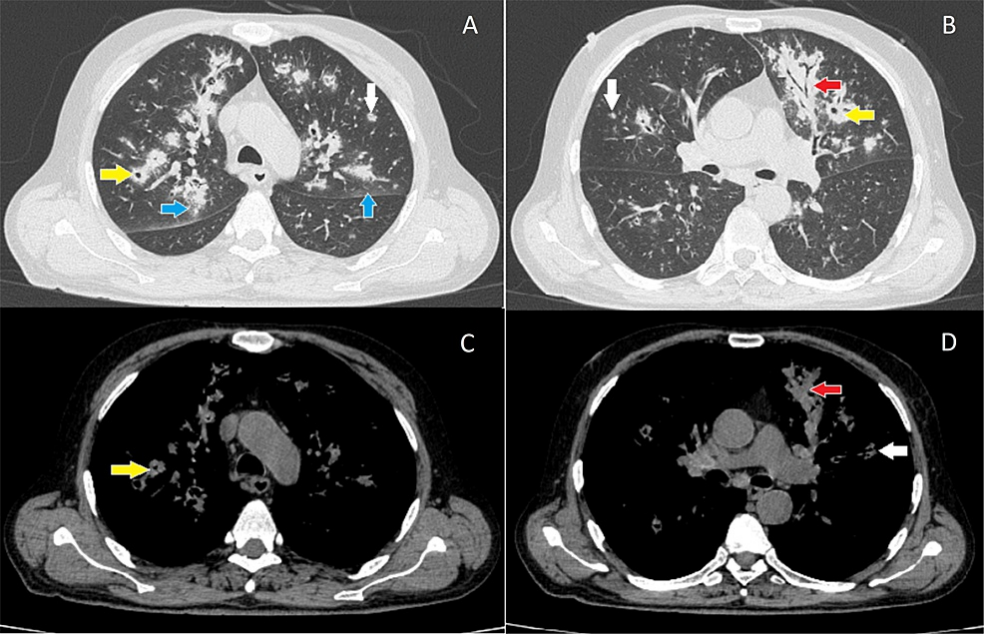
Computed tomography of the chest; lung (A and B) and mediastinal windows (C and D). Multifocal cavitating consolidations (yellow arrows: A, B, C) involving both lung fields with surrounding ground glass halos (blue arrows: A). Consolidation with air bronchograms is seen in the left lung (red arrow: B, D). Multiple ground glass nodules scattered randomly in both lung fields (white arrows: A, D).

Chest x-ray revealed bilateral infiltrates, confirmed by high-resolution computed tomography (Figure 3, Figure 4).

Given the new onset of confusion without an obvious cause, a computed tomography of the brain and a lumbar puncture were performed on the second day of admission which were normal. Keeping in view the symptomatic thrombocytopenia, potential exposure to animals during the Eid ul Adha festival and the infiltrates on the chest x-ray, samples were sent to the provincial reference laboratory on the second day of hospitalization for the detection of dengue virus, CCHF virus and SARS-CoV-2 ribonucleic acid by polymerase chain reaction (PCR). The PCRs, reported on day four of admission, were positive for all three viruses. Screening tests for atypical pneumonia and leptospirosis were negative. Sputum was collected for bacterial culture and sensitivity, and acid-fast bacilli (AFB) staining, gene Xpert, and culture. There was no growth from the sputum, and AFB staining and gene Xpert were negative. Bronchoscopy and bronchoalveolar lavage were planned. 

A multidisciplinary team recommended treating him conservatively and ribavirin was added to his treatment regimen. Despite the supportive treatment, antibiotics, and ribavirin, he developed multi-organ failure (Table 2).

**Table 2:** Laboratory investigations during the hospital stay. g/dL: Gram/deciliter, mcL: Microliter, mg/dL: milligram/deciliter, IU/L: International unit/liter

Investigations	Reference range	28.7.22	30.7.22	01.08.22	03.8.22	06.08.22	08.08.22
Hemoglobin (g/dL)	13.5 – 17.5	9.1	9.0	9.1	8.2	7.9	7.7
Platelet count (x10^3^/mcL)	150 – 450	22	34	20	18	23	17
White cell count (x10^3^/mcL)	4.5 – 11	8.6	8.5	3.2	9.8	13.5	18.5
Neutrophils (%)	40 – 60 %	78	85	73	81	88	88
Lymphocytes (%)	20 – 40 %	13	11	22	16	06	04
Monocytes (%)	2 – 8 %	08	04	05	03	06	07
Eosinophils (%)	1 – 4 %	01	00	00	00	00	01
Total bilirubin (mg/dL)	0.2 – 1.2	0.7	0.9	1.0	1.3	1.9	2.3
Alanine aminotransferase (IU/L)	< 45	244	231	298	341	334	349
Alkaline phosphatase (IU/L)	30 – 120	110	112	127	138	151	156
Urea (mg/dL)	18 – 45	82	89	96	119	214	228
Creatinine (mg/dL)	0.6 – 1.2	1.3	1.4	1.8	2.4	4.0	4.9
C-reactive protein (mg/dL)	< 0.5	34.8	27.8	25	34.9	23	25.6

On day 12 of admission, he had a sudden cardiac arrest with asystole and could not be revived. The case was reported to the public health authorities.

## Discussion

Until 15 July 2022, 1,544,131 COVID-19 cases and 30426 deaths have been reported in Pakistan [10]. Amidst this pandemic, the National Institute of Health (NIH) in Pakistan issued a ‘High Alert’ for dengue viral infection during the monsoon season (July-December) and CCHF around the Eid ul Adha (July 2022) to quell the increasing risks of infection [11].

Nature is full of surprises and can present in any form and combination of infections. This indexed case presented with triple co-infection of Dengue virus, CCHF virus, and SARS CoV-2 virus, which has never been reported before. He was treated with antibiotics, blood products, ribavirin, and supplemental oxygen, but did not survive. Büyüktuna et al. and Dülger from Turkey have reported two cases of COVID-19 co-infection in a CCHF patient. The latter was treated with favipiravir and survived [12,13].

Yan et al. from Singapore have found a false positive Dengue NS-1 in two COVID-19 patients and urged PCR confirmation [14]. A systematic review by Tshetenet et al. found 13 cases of co-infection of COVID-19 and dengue fever confirmed on PCR [15]. This case had dengue fever, CCHF and COVID-19 confirmed by PCR.

Dengue virus acts through cellular receptors in human monocytes and macrophage cells via the envelope (E-protein) of the virus [16]. The CCHF virus interacts with the endothelial cells directly, and it also interacts indirectly via immune cells with the subsequent release of proinflammatory cytokines [17]. The SARS-CoV-2 infection enters the host cells through the S spike protein by binding to ACE2 receptors for internalization, causing immune modulation and cytokine storm [18]. Keeping in mind the different pathophysiologic mechanisms of all three viruses in a single case, it would be very challenging to understand the exact host response, virus interplay, and corresponding treatment.

This case will act as an eye opener for clinicians, epidemiologists, virologists, and local and international public health authorities to forecast and tackle the nexus of different pathogens to quell the emergence of a new co-epidemic in this region.

## Conclusions

Though co-infections are uncommon, these are on the rise due to the changing socio-demographic risk factors. Because of the difficulty in diagnosis and treatment, co-infections have higher mortality. Despite different modes of transmission, and predilection for different target organs but common socio-cultural and epidemiological risk factors, the presence of triple co-infection, in this case, should act as a red signal to alert the regional and international public health authorities. Co-infections should not be taken for granted as these are not merely regional issues but may threaten global health.
